# The PEG-coated collagen patch Hemopatch® for hemostasis and dural sealing in neurosurgery

**DOI:** 10.3389/fsurg.2025.1636372

**Published:** 2025-08-20

**Authors:** Jakob Rossmann, Apio C. M. Antunes, Guy G. Broc, Danny T. M. Chan, Se-Yi Chen, Nicolas Dea, Jacob Fairhall, Pablo de Andrés Guijarro, Nicola Montano, Karl-Michael Schebesch, Jake Timothy, Vamsi Krishna Yerramneni

**Affiliations:** ^1^ Department of Neurosurgery, Paracelsus Medical University, Nürnberg, Germany; ^2^ Neurosurgery Department, Universidade Federal do Rio Grande do Sul and Hospital de Clínicas de Porto Alegre, Porto Alegre, Brazil; ^3^ Neurosurgery Department, American British Cowdray Hospital, Mexico City, Mexico; ^4^ Division of Neurosurgery, Department of Surgery, Prince of Wales Hospital, The Chinese University of Hong Kong, Hong Kong, Hong Kong SAR, China; ^5^ Department of Neurosurgery, Chung Shan Medical University Hospital, Taichung City, Taiwan; ^6^ Division of Neurosurgery, Department of Surgery, Vancouver General Hospital and the University of British Columbia, Vancouver, BC, Canada; ^7^ BrainSpine Neurosurgery, Prince of Wales Hospital, Randwick, NSW, Australia; ^8^ Neurosurgery, Hospital Universitario Fundación Jiménez Díaz, Madrid, Spain; ^9^ Department of Neurosurgery, Fondazione Policlinico Universitario Agostino Gemelli IRCCS—Università Cattolica del Sacro Cuore, Rome, Italy; ^10^ Department of Neurosurgery, Nuffield Hospital, Leeds, United Kingdom; ^11^ Department of Neurosurgery, Nizam’s Institute of Medical Sciences, Hyderabad, India

**Keywords:** dural sealant, hemostasis, spinal, CSF leakage, neurosurgery, cranial

## Abstract

**Background:**

Postoperative cerebrospinal fluid (CSF) leakage and bleeding are major postoperative complications that increase healthcare system costs. The use of Hemopatch® Sealing Hemostat has been shown to reduce the incidence of such postoperative complications. This technical report aims to provide neurosurgeons with the best recommendations for the effective use of Hemopatch® as a hemostatic and dural sealant in cranial and spinal procedures.

**Material and methods:**

The clinical experiences of 10 neurosurgeons from around the world regarding the use of Hemopatch® were evaluated using an online survey, followed by a hands-on preclinical workshop on adult pigs, which concluded with an in-depth discussion about the use of the patch.

**Results:**

The survey results provide an overview of how and when experts use different types of dural repair materials, including decision-making factors. During the workshop, Hemopatch® presented excellent tissue adherence on all evaluated defects. The new configuration of the patch showed improved tissue adherence, less curling of the patch, and easier removal of the gauze used for compression. Experts recommend using patches that overlap the defect for ≥1 cm. When closing defects that do not allow for a dried application site, Hemopatch® can be put on a dry gauze, which can be bent into a U-shape. This allows better targeting of the application site and enables immediate compression upon placement.

**Conclusion:**

The results provide information to improve the hands-on use of Hemopatch® as a dural sealant, therefore reducing the risk of postoperative complications such as CSF leaks, eventually reducing healthcare system costs.

## Introduction

Rigorous hemostasis and closure of the dura are basic principles in neurosurgery. In addition to traditional surgical techniques such as suture, bipolar coagulation, hydrogen peroxide, and many more, a wide variety of adjunctive hemostatic products are used to achieve hemostasis (1). Similarly, watertight dural closure is achieved not only with autologous tissue but also with commercially available dural substitutes and sealants (2). A variety of fluid- and fleece-bound dural sealants are available and have been thoroughly tested for their efficacy and safety in recent years (2–6).

Postoperative cerebrospinal fluid (CSF) leakage is a major postoperative complication that puts patients at a higher risk of infection, the need for revision surgery, and thus prolonged hospital stay (7, 8). These outcomes significantly increase healthcare system costs.

Hemopatch® Sealing Hemostat is a polyethylene glycol (PEG)-coated collagen fleece that is indicated for hemostasis and sealing. Its efficacy in neurosurgery has been well-described to reduce the incidence of postoperative CSF leaks when used as a dural sealant (9–17). In a study by Sanchez Fernandez and Rodriguez-Arias, 230 patients who underwent cranial procedures were retrospectively analyzed. The incidence of CSF leaks was significantly reduced in patients who received the PEG-coated collagen patch compared with the control group that received standard of care only (7.8% with Hemopatch® vs. 30.4% in the control group, *P* < 0.001) (15). In another prospective, multicenter, single-arm observational study, Hemopatch® was used in 147 patients who underwent a cranial or spinal procedure. The results indicated a safe and effective use of the PEG-coated collagen patch at the dura, with 93% postoperative water tightness (no CSF leakage) achieved (17). Low CSF leakage rates of 0%, 4.54%, and 5.9% after the use of the PEG-coated collagen patch were described in early patient series (9, 10, 12). The use of sealants has been shown to reduce hospital costs by reducing CSF leakage rates (18, 19). Given the successful use of Hemopatch® as a dural sealant, defining a standardized application protocol will encourage its correct use and may further improve patient outcomes. This technical report aims to provide neurosurgeons with the best recommendations for the successful use of the PEG-coated collagen patch and a new, upcoming configuration of the product (referred to as “Hemopatch RT” in this article), as a hemostatic and dural sealant in cranial and spinal procedures, and to come closer to developing a standardized application protocol.

## Materials and methods

The clinical experiences of 10 neurosurgeons from around the world (Australia, Brazil, Canada, China, Germany, India, Mexico, Spain, Taiwan, and the UK) regarding the use of Hemopatch® were evaluated using an online survey. This survey was followed by a hands-on preclinical workshop held on 3–5 November 2023 in Bad Saarow, Germany, and concluded with an in-depth discussion on the use of the PEG-coated collagen patch as a dural sealant and hemostat in cranial and spinal neurosurgery.

### Pre-meeting online survey

Before the start of the workshop, a pre-meeting survey about the incidences of CSF leaks and established surgical practices in dural closure procedures was sent to all 10 participating neurosurgeons. All participants answered the survey before the start of the meeting without being aware of other participants' answers. A consensus on a statement was assumed when ≥70% of participants responded similarly.

### Hemopatch® mechanism of action

Hemopatch® Sealing Hemostat was developed and is distributed by Baxter Healthcare SA [8152 Glattpark (Opfikon), Switzerland] as a Class III medical device in many countries worldwide. It was first launched in October 2013 with a hemostasis indication. Sealing indications, including dural sealing, were added to the label in the fourth quarter of 2016.

Hemopatch® consists of a collagen patch that is coated with PEG. A detailed description of its composition, properties, and mode of action was published earlier (20). When the patch comes in contact with blood or other body fluids, the PEG coating on the non-marked, white side dissolves and starts to cross-link. The reactive PEG groups form covalent bonds with free amine groups on the tissue, e.g., amino acid residues, forming a hydrogel which firmly attaches the patch to the underlying tissue, sealing the defect. As sealing of a bleeding source with PEG hydrogel is independent of the patient’s coagulation status, the product works even in patients with a compromised coagulation status, as shown in preclinical and clinical studies (20–23). The non-coated side of the patch is marked with small blue squares to better distinguish it from the coated side (“blue to you”). These marks also provide orientation when smaller pieces of the patch need to be cut. Detailed recommendations for the optimal application of the PEG-coated collagen patch can be found in the Results section.

### Hands-on preclinical workshop

A live tissue surgery was performed on pigs under an animal experiment permission granted by the respective authority in Brandenburg (Landesamt für Arbeitsschutz, Verbraucherschutz und Gesundheit).

Two young adult pigs with an approximately 35–40 kg body weight were anesthetized by intramuscular injection of standard anesthetics. After 15 min, an ear vein access was placed, and 5 mg/kg propofol was injected for analgesia. The animals were then endotracheally intubated and mechanically ventilated. Balanced anesthesia was maintained with inhalation anesthesia. Throughout the procedure, the animals were monitored for heart rate, blood pressure, airway pressure, and body temperature and provided with anesthetics and analgesics as needed. Qualified veterinary staff were present throughout the procedure. After completion of all procedures, the animals were euthanized under deep anesthesia with a lethal dose of thiopental sodium and embutramide/mebezonium iodine/tetracaine hydrochloride (T61; Hoechst Marion Roussel, Brussels, Belgium). The established anesthesia management of pigs is described in greater detail elsewhere (24).

In the practical part, 10 neurosurgeons (AA, GB, DC, S-YC, ND, JF, PA, JR, JT, VY) used Hemopatch® as primary sealant in different challenging situations on the anesthetized live pigs to evaluate the best application techniques during a surgical skills training session. The animals were placed in a prone position, and for the cranial procedures, the head was shaved and sterilized, and the skin was opened. A midline parietal craniectomy was performed using a cranial drill. For the spinal procedures, the back was shaved and sterilized, and the skin along the midline was opened. Soft tissue surrounding the vertebrae was removed with an electrocautery knife. To expose the spinal cord, a laminectomy was performed. The surgeons tried to mimic typical intraoperative situations by creating defects, leading to bleeding or CSF leakage by using a scalpel and/or microhook. When feasible, bleeding grades were rated according to the VIBe scale before treatment (25). Based on the low number of defects and the difficulty to exactly reproduce defects, no statistical evaluation could be performed, but results were evaluated based on consensus among the surgeons regarding their observations.

In addition to the already marketed Hemopatch®, which is storable at room temperature for 6 months, a new “Hemopatch RT” configuration, provided as an R&D sample, with an extended shelf life of 36 months at room temperature (RT, based on a new packaging configuration), was tested by all neurosurgeons. The neurosurgeons were blinded to the configuration of the patch provided. The products were applied following the instructions for use (IFU). The target application area was dried from excess liquid using dry gauze. Patches were held in place with a dry gauze or sponge with appropriate compression for 2 min, before carefully removing the gauze and inspecting the application area for successful hemostasis or sealing. To facilitate the removal of the gauze in cases where it adhered to the patch, gentle irrigation with saline solution could be applied.

### Roundtable expert discussion

Concluding the workshop, a follow-up discussion was held, at which all 10 attending neurosurgeons discussed their experience with Hemopatch® from previous clinical use as well as the practical preclinical testing in the workshop. The results of the survey were discussed, and the suitability of the PEG-coated collagen patch for addressing challenging situations in neurosurgery was critically evaluated. Additionally, observations during the practical application of Hemopatch® and “Hemopatch RT” were discussed.

## Results

### Pre-meeting survey

#### CSF leakage

Experts agreed that the incidence of CSF leaks after neurosurgical procedures with dural closure is usually below 10% and can be further reduced by minimally invasive surgery approaches (Table 1).

**Table 1:** CSF leakage rates according to surgical approach and anatomical location.

Incidence of CSF leaks after open surgical approaches					
Range	<10%	11%–20%	21%–30%	31%–40%	>40%
Responses					
Supratentorial	100%				
Posterior fossa	70%	20%	10%		
Skull base surgeries	70%	10%	20%		
Cervical spine surgeries	100%				
Thoracolumbar surgeries	90%	10%			
Incidence of CSF leaks after MIS surgical approaches					
Range	<10%	11%–20%	21%–30%	31%–40%	>40%
Responses					
Supratentorial	100%				
Posterior fossa	80%	10%	10%		
Skull base surgeries	80%	20%			
Cervical spine surgeries	100%				
Thoracolumbar surgeries	100%				
Is the gap size after primary dural closure a decision-making factor for the choice of dural repair material?					
Yes	90%				
No	10%				

MIS, minimally invasive surgery.

More than one expert named the following surgeries as being of high risk for the development of a CFS leakage: posterior fossa (with high intracranial pressure), (endoscopic or anterior) skull base, revision, and spine surgery. Additional surgeries that were mentioned only once included severe lumbar canal stenosis; endonasal/transsphenoidal, bifrontal, or suboccipital decompressive craniectomy; and vertebral fusion surgery (spondylodesis).

More than one expert named the following patient profiles as being of high risk for the development of a CSF leak: advanced age, obesity, postradiation therapy status, meningioma, or synovial cyst. Additional patient profiles that were mentioned only once included ossification of the posterior longitudinal ligament (OPLL), ossification of the ligamentum flavum (OLF), poor wound healing status, pediatric spinal dysraphism, and ventricular cavity opening to the subarachnoid spaces.

**Figure 1:**
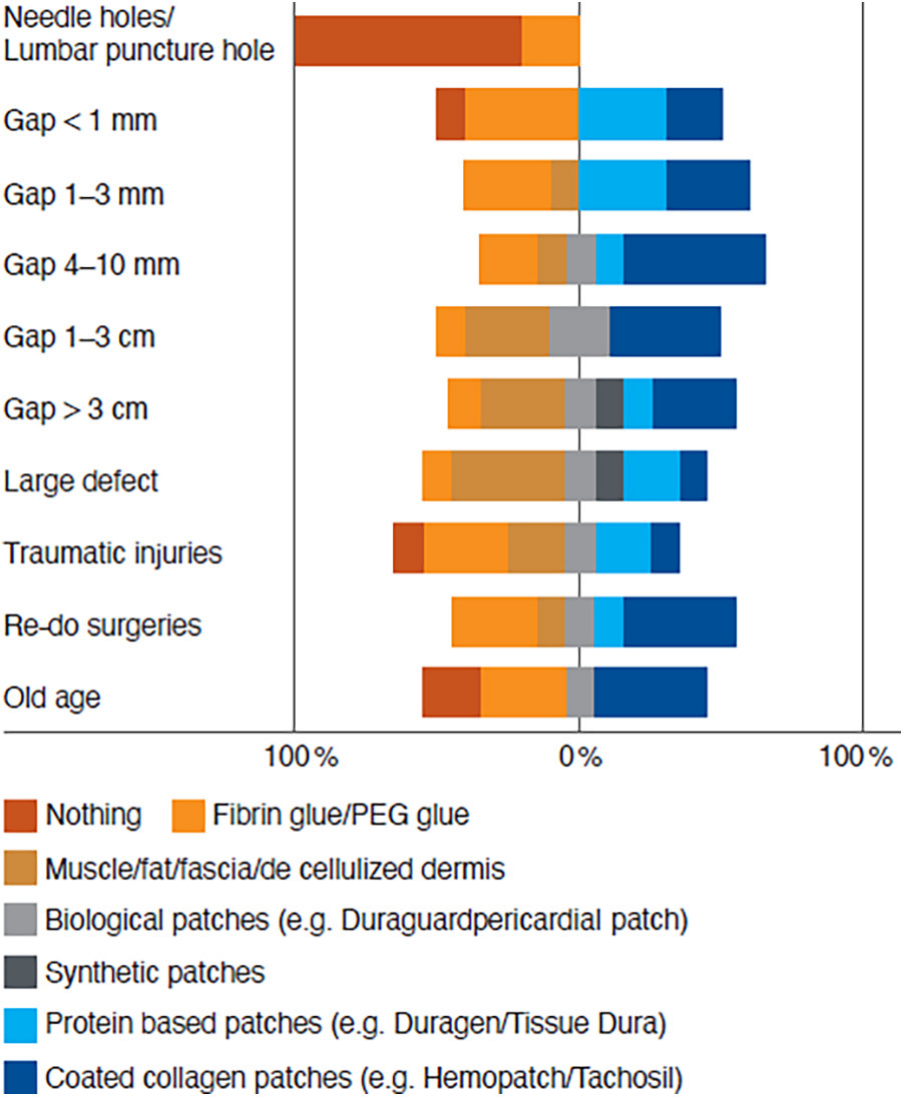
Established repairs of different dural defects. Experts were asked how they generally approach the different dural defects.

Experts agreed that dural defects of the size of a needle or lumbar puncture hole do not require an additional sealant for closure. Only a minority of experts indicated they would use a fibrin or PEG sealant to close such a small hole. Experts had different opinions about the approach for closing gaps of 1–3, 4–10, 1–3, and >3 cm, or large defects, and revision surgeries. In all cases, approaches varied from fibrin/PEG sealant over muscle/fat/fascia/decellularized dermis, biological patches (except 1–3 mm gaps), synthetic patches (>3 cm or large defects only), protein-based patches to coated collagen patches. There was further difference of opinion among experts regarding approaches for closing traumatic injuries or dural defects in older patients. In both cases, entries ranged from doing nothing, extending to fibrin/PEG sealant, muscle/fat/fascia/ decellularized dermis, biological patches, protein-based patches to coated collagen patches (Figure 1).

Experts agreed that the gap size after primary dural closure is a decision-making factor for the choice of the dural repair material (Table 1; Figure 1).

#### Use of dural substitutes and dural sealants

Experts agreed that they routinely perform primary dural closure by suturing. Only one neurosurgeon used a vascular closure stapler (VCS clips) instead. One neurosurgeon mentioned that there is a trend toward the use of biological dural substitutes. Experts further agreed that they prefer to add sealant, such as glue or a patch.

Experts agreed that they decide whether a patient requires additional measures for suturing the dura, depending on the surface structure of the dural closure, the time needed for closure, and the size of the defect. It was additionally mentioned that the methodological approach depends more on anatomical than patient factors.

Approximately 50% of experts preferred a patch as a dural sealant over a liquid/gel type. Other experts mentioned using liquid gels for small gaps or slits and patches for larger defects.

Experts agreed that based on the dural defect size, they decide whether a patient requires a dural sealant or a dural substitute. Most experts used dural sealant on smaller size defects and dural substitutes or a combination of both on larger size defects.

Experts disagreed on the use rate of dural sealant and dural substitute. For both methods, an evenly distributed rate of <20% to >80% was mentioned.

Experts disagreed on the percentage of cases where they use both a dural sealant and a dural substitute. Most experts answered with <20%, but the remaining responses were evenly distributed (20%–40%, 40%–60%, 60%–80%, and >80%).

More than one expert preferred using dural sealant in combination with a dural substitute in the following surgical situations: (endoscopic) skull base and posterior fossa surgery (with high intracranial pressure). Additional surgical situations that were mentioned only once included transsphenoidal surgery, post-meningioma excision, and spinal dysraphism repair surgery, as well as surgeries with large dural defects or dural tears.

More than one expert used a patch with both hemostatic and sealing properties in the following indications: posterior fossa and (endoscopic or anterior) skull base surgery. Additionally, indications that were mentioned only once included iliac vein tears, parietal region surgeries, spinal epidural space surgeries, cranial surgery, glioma surgeries, and dural openings at the side of a venous sinus. An overview of the pre-meeting results is provided in Table 2.

**Table 2:** Pre-meeting survey results.

Experts agreed…	Experts disagreed…
…on the incidence of CSF leaks after neurosurgical procedures	…on the use rate of dural sealant and dural substitute
…that dural defects of the size of a needle or lumbar puncture hole do not require closing	…on the percentage of cases where they use both dural sealant and dural substitute
…that the gap size after primary dural closure is a decision-making factor for the choice of the dural repair material	
…that primary dural closure is performed by suturing	
…on parameters to decide whether a patient requires additional measures for dural suturing	
…that dural defect size is a decision-making factor whether a patient requires a dural sealant or a dural substitute	

### Hands-on preclinical workshop

#### Cranial defects

Bone bleeding of Grade 1–2, based on the VIBe intraoperative bleeding scale (25), a small venous bleeding, an epidural edge bleeding, and two different sagittal sinus bleedings were sequentially induced using a microhook and/or scalpel.

Observations with the use of “Hemopatch RT,” as the new configuration, are summarized below. Bone bleeding was controlled, the patch adhered well to the bone surface, and the gauze was easily removed after 2 min approximation.

The small venous bleeding was stopped as well, and the surgeons agreed that the patch showed an excellent adherence to the tissue after removal of the approximating gauze. Similarly, epidural edge bleeding was stopped effectively, indicating excellent tissue adherence of “Hemopatch RT” after easy removal of the approximating gauze.

The first sinus bleeding was treated using “Hemopatch RT,” which was placed on a bent gauze to ensure immediate approximation and compression upon placement, as the high flow of blood created a wet application area. After 2 min of compression with the gauze, a small bleeding was still observed, as the patch did not completely cover the defect.

The second sinus bleeding was classified as VIBe grade 3, and “Hemopatch RT” stopped the bleeding effectively and showed excellent tissue adherence after easy removal of the gauze.

A CSF leakage was then induced by opening the dura with a microhook. “Hemopatch RT” was applied for 2 min. The gauze used for compression was easy to remove, and the patch showed excellent tissue adherence and achieved sealing of the CSF leak.

With the PEG-coated collagen patch recently marketed, similar observations were made, but in some cases, the approximating gauze was not very easy to remove, and some saline solution was used to facilitate removal. In a few cases, some curling of the patch at the edges was observed.

#### Spinal defects

An intradural bleeding, an epidural venous ooze of VIBe Grade 2, and a CSF leak were sequentially induced with a microhook and/or scalpel.

The intradural bleeding induced by opening the dura was stopped by applying “Hemopatch RT,” and after 2 min of compression, an excellent tissue adherence of the patch was observed.

The epidural venous ooze was stopped immediately with “Hemopatch RT,” and the adherence of this patch after 2 min approximation was excellent.

The spinal CSF leak was successfully sealed with “Hemopatch RT.” The patch adhered well to the dura, and the gauze used for compression was easy to remove. Applications of Hemopatch® to these defects led to similar observations, and in one case, some curling at the edges of the patch was observed.

### Final expert discussion

After completion of the workshop, the team of neurosurgeons discussed the results of the pre-meeting survey and their experiences during the workshop. The results of the survey, described above, were discussed, underlining the findings that for the closure of larger defects, many autologous and commercial materials can be used, based on the availability and preference of the surgeon, and that sealants and sealing patches can be used alone or in combination with dural substitutes to finally achieve watertight dural closure. Recommendations regarding the best use of Hemopatch® are summarized below.

#### Recommendations for Hemopatch® application

If the PEG-coated collagen patch needs to be cut to a specific size, it must be cut large enough to overlap the defect by 1 cm in each direction to achieve the best results.

**Figure 2:**
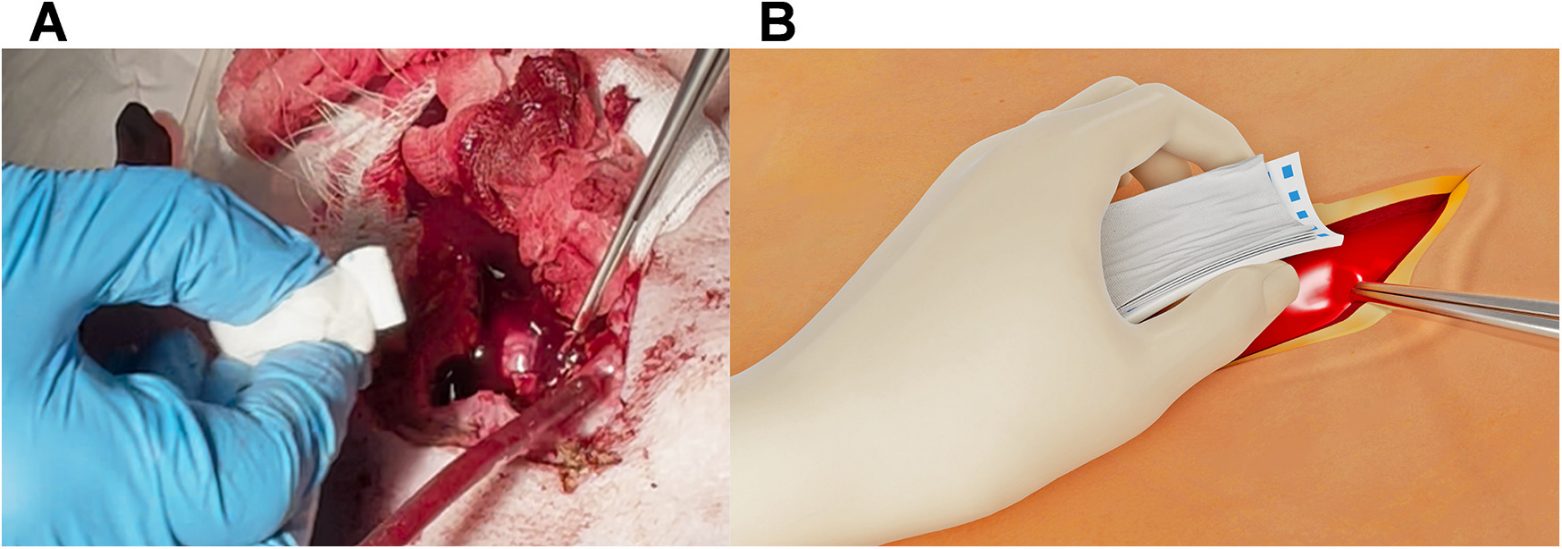
Hemopatch® placed on a gauze to allow immediate approximation and compression **(A)** and a schematic drawing of the principle **(B)**.

If the flow of body fluid (blood or CSF) is high and does not allow for a dried application site, the PEG-coated collagen patch can be put on a dry gauze, which can be bent to a U-shape with the fingers. This allows for better targeting of the application site and enables immediate approximation and compression upon placement (Figure 2).

#### Properties of “Hemopatch RT”

During the workshop, all neurosurgeons were able to evaluate the new configuration “Hemopatch RT” and compare its characteristics with the currently marketed Hemopatch® in different neurosurgical settings. As the number of applications was limited, no statistically valid evaluation could be performed, but according to the surgeons’ perception, “Hemopatch RT” showed an excellent adherence to the tissue, and the approximating gauze could be easily removed from the patch. Overall, neurosurgeons agreed that they would welcome the market introduction of “Hemopatch RT” as it performed very well in their hands.

## Discussion

This report presents the results of a survey, workshop, and expert discussion on the proper use and applications of the hemostatic and dural sealant Hemopatch® in neurosurgery.

### Hemopatch® as a dural sealant during neurosurgery

Hemopatch® is licensed as a surgical sealant for procedures in which control of body fluids by conventional surgical techniques is either ineffective or impractical. Furthermore, it is not intended as a substitute for meticulous surgical techniques and the proper application of ligatures or other conventional procedures for sealing (26). Since its market introduction as a dural sealant in 2016, Hemopatch® has been therefore used as a watertight primary sealant for smaller CSF leaks or dural defects and as a secondary sealant on larger sutured dural defects in various neurosurgical procedures (9, 10, 12, 14, 15, 17). During the neurosurgical procedures performed in the workshop by the 10 neurosurgeons, the PEG-coated collagen patch was used only as a primary sealant to evaluate its efficacy in more exceptional circumstances. It was successfully used to control bleeding and CSF leakages in cranial and spinal scenarios. The efficacy of Hemopatch® as a dural sealant for cranial and spinal surgeries was already validated in four prospective, single-center studies (9, 12–14) and one prospective multicenter, single-arm observational study (17). Furthermore, it was evaluated for cranial approaches in a retrospective, single-center cohort study (15) and by Schebesch and Brawanski in supratentorial, infratentorial, and transsphenoidal approaches. The authors concluded in their retrospective clinical experience report that the use of the PEG-coated collagen patch for these approaches is safe and feasible (10). Based on the experts' discussion, there seem to be only minor differences in the use of Hemopatch® for cranial and spinal surgery. However, experts also mentioned that cranial surgeries usually require a suture for primary dural closure and dural patches should be used as a secondary sealant to achieve a watertight closure, while in spinal surgeries, it might be sufficient to use a sealing patch as a primary sealant. Similar results were obtained by Montano and colleagues (12) who mentioned that it is often not possible to close incidental spinal durotomies by suturing. In these cases, dural patches might be used as a primary sealant. Moreover, dural sealants have also been shown to reduce epidural fibrosis in spinal surgery (27, 28). Two fluid dural sealants proved to be safe and effective in reducing CSF leaks in over 90% of patients in a large prospective study with 250 patients (6).

Intentional durotomies, irrespective of whether spinal or cranial, are more likely to require suturing for primary closure, followed by a sealing patch as secondary sealant. Experts assume that suturing will become less common in the future. In a recent publication, researchers even used Hemopatch® as a dural substitute (29). As this application is outside the intended use by the manufacturer, the expert group recommended using it as a dural sealant only, as this is shown to be safe.

Experts also discussed which applications of Hemopatch® they see, in cases with a high risk of CSF leakage, and in which they prefer to use a dural sealant in combination with a dural substitute. The most relevant answers to these two questions were identical, suggesting that experts indeed use a dural substitute in combination with a dural sealant in situations that carry a high risk of a CSF leak. Some of the applications mentioned match the already published indications for the use of the PEG-coated collagen patch, such as posterior fossa (10, 13–15) and skull base surgeries (15).

In the survey, experts were also asked for patient profiles that are at high risk for the development of a CSF leak. More than one expert cited advanced age, obesity, postradiation therapy status, meningioma (especially with dural excision), or synovial cysts as risk factors for postoperative CSF leakage. Such risk factors are the subject of controversial debate in recent research. A few of these factors are shown to influence the CSF leakage rate in some but not all applications, while others do not (30–33). A recent study by Atchley and colleagues (34) developed a predictive model and clinical risk score for postoperative CSF-related complications after posterior fossa and posterolateral skull base surgeries and therefore applications that were also repeatedly mentioned by the experts. The authors based their highly specific risk score on the factors age, body mass index, dural repair technique, use of bone substitute, and duration of postoperative CSF diversion, suggesting that at least patients' age and weight might indeed influence the risk for the development of postoperative CSF leakage.

Overall, the group of experts confirmed that Hemopatch® is well-suited as a dural sealant when applied following the instructions.

## Strengths and limitations

The most relevant strength of our results lies in the 10 expert neurosurgeons who shared their skills and knowledge regarding the use of Hemopatch® from all over the world and from a wide variety of neurosurgical approaches. Limitations of our report are to be found in the preclinical method and in the non-standardized approach of comparing Hemopatch® and “Hemopatch RT” during the workshop. The recommendations in this work are expert consensus opinions. Standardized clinical tests are needed to validate these experts’ recommendations and opinions.

## Conclusion

The results of our workshop validate the efficacy of Hemopatch® as a hemostatic sealant for neurosurgical procedures. The results of the survey, workshop, and expert discussion provide detailed information on the state-of-the-art use of Hemopatch® for cranial and spinal dural sealing. Although experts did not agree on all questions, a certain level of consensus could be reached, and therefore, a closer approach to a more standardized application protocol could be achieved. The results provide information to improve the hands-on use of Hemopatch® as a dural sealant, therefore reducing the risk of postoperative complications such as CSF leaks and eventually reducing the healthcare system costs. Further research is needed to define the most appropriate dural closure method and materials to reach the best patient outcome possible.

## Data Availability

The raw data supporting the conclusions of this article will be made available by the authors, without undue reservation.
